# The effect of temperature on dengue virus transmission by *Aedes* mosquitoes

**DOI:** 10.3389/fcimb.2023.1242173

**Published:** 2023-09-21

**Authors:** Zhuanzhuan Liu, Qingxin Zhang, Liya Li, Junjie He, Jinyang Guo, Zichen Wang, Yige Huang, Zimeng Xi, Fei Yuan, Yiji Li, Tingting Li

**Affiliations:** ^1^ Department of Pathogen Biology, Center for Tropical Disease Control and Research, School of Basic Medical Sciences and Life Sciences, Key Laboratory of Tropical Translational Medicine of Ministry of Education, Hainan Medical University, Haikou, China; ^2^ Department of Pathogen Biology and Immunology, Jiangsu International Laboratory of Immunity and Metabolism, Jiangsu Key Laboratory of Immunity and Metabolism, Xuzhou Medical University, Xuzhou, China; ^3^ School of Imaging Medical Sciences, Xuzhou Medical University, Xuzhou, China

**Keywords:** temperature, dengue virus, *Aedes albopictus*, *Aedes aegypti*, vector competence

## Abstract

Dengue is prevalent in tropical and subtropical regions. As an arbovirus disease, it is mainly transmitted by *Aedes aegypti* and *Aedes albopictus*. According to the previous studies, temperature is closely related to the survival of *Aedes* mosquitoes, the proliferation of dengue virus (DENV) and the vector competence of *Aedes* to transmit DENV. This review describes the correlations between temperature and dengue epidemics, and explores the potential reasons including the distribution and development of *Aedes* mosquitoes, the structure of DENV, and the vector competence of *Aedes* mosquitoes. In addition, the immune and metabolic mechanism are discussed on how temperature affects the vector competence of *Aedes* mosquitoes to transmit DENV.

## Introduction

Dengue is an acute infectious disease caused by dengue virus (DENV), which is transmitted by *Aedes aegypti* (*Ae.aegypti*) and *Aedes albopictus* (*Ae.albopictus*) (Bifani et al., 2022). Dengue is characterized by rapid transmission, high morbidity, universal susceptibility and high fatality rate. The majority of the infected are asymptomatic or only experience mild symptoms including high fever, headache, skin rash, and systemic muscle and joint aches. A few cases may develop severe bleeding and other clinical complications, such as skin purpura and ecchymosis, nosebleed, digestive and urogenital tracts bleeding, hemorrhagic shock, which may even lead to death (Gulati and Maheshwari, 2007; Wilder-Smith et al., 2019). The severe dengue is more common among infants, old people, pregnant individuals, people experiencing a second dengue infection, and people with certain underlying conditions (Hernandez-Romieu et al., 2023).

Dengue is a growing problem in its geographical spread (Brady et al., 2012) and has established its own status globally in both endemic and epidemic transmission cycles (Bhatt et al., 2013). It is estimated that 390 million people from over 125 countries are annually infected with dengue (Gutierrez-Barbosa et al., 2020). Dengue is mainly prevalent in tropical and subtropical regions, especially in Africa, South America, South Asia, Southeast Asia, and the Western Pacific region (Wilder-Smith et al., 2019). In 2016, the largest dengue outbreak occurred in Cordova, Argentina (Rotela et al., 2017). The number of cases in Latin America also increased dramatically in recent years, and the major outbreak was reported in 2019, with a total of 3,140,872 cases (Gutierrez-Barbosa et al., 2020). Colombia experienced its fifth dengue outbreak in 2019 (Cousins, 2019). The same year, Honduras reported the worst local dengue outbreak in the past five decades, with a total of 342,346 cases (Dos et al., 2019). In America, more than 30,000 local-acquired and 7000 travel-associated dengue cases were reported from 2010 to 2021 (Hernandez-Romieu et al., 2023). In Thailand, dengue cases increased with a yearly average of 91,650 cases between 2009 and 2015, with the peak year in 2013 of 154,000 total dengue cases and 156 deaths (Tangsathapornpong and Thisyakorn, 2023). There were 81,653 indigenous dengue cases reported in mainland China from 2005 to 2020, across 345 counties in 14 provinces and municipalities (Yue et al., 2022). Provinces of China reporting annual dengue cases have expanded from the southeast coast to the southwest, central, northeast, and northwest regions, with higher incidence in Guangdong, Guangxi, Yunnan, Fujian, Zhejiang Province (Lin et al., 2020).

The World Health Organization (WHO) acknowledged that health issues related to climate change are among the most significant challenges in the 21st century, with dengue ranking at the top of their concerns (http://www.who.int/globalchange/health_policy/en/). The transmission of dengue is greatly influenced by temperature. By effectively mitigating global warming, a substantial reduction in the number of dengue cases can be achieved. The Paris Climate Agreement sets the objective of limiting the global average temperature increase to within 1.5°C since the pre-industrial period. Consequently, it is projected that the number of dengue cases will decrease by 300,000 per year by 2050 and by 500,000 per year by 2100. Additionally, by curbing global warming the spread of dengue to areas with lower incidence rates could also be prevented (Friedrich, 2017).

This review intends to discuss the impact of temperature on the transmission of the dengue virus by *Aedes* mosquitoes. Based on model predictions and analyses, the correlation between temperature and the prevalence and distribution of dengue cases will be elucidated. Furthermore, three key aspects will be explored to investigate potential causes and mechanisms. (i) the impact of temperature on the survival and distribution of *Aedes* mosquitoes; (ii) how temperature alters the structure of DENV; (iii) the effect of temperature on the vector competence of *Aedes* mosquitoes in transmitting DENV. This review will serve as a valuable resource for the prevention and control of dengue.

## Temperature affects the prevalence of dengue

Abundant studies indicated that temperature was positively correlated with dengue cases (Fan et al., 2014). The most suitable minimum temperature for the transmission of DENV is 14.8°C (Feldstein et al., 2015), while the optimal maximum temperature ranges from 32°C to 33°C (Stephenson et al., 2022). Generalized additive models (GAMs) were employed to study the factors influencing the spread of dengue. The equation of this model is defined as $\text{g}\left(\text{E}\left(\text{Y}\right)\right)=\text{α}+\sum_{\text{i}=1}^{\text{k}}\text{S}\left(\text{X},\text{d}\right)$ , where Y represents the number of dengue cases, α is a constant term, S denotes a non-linear smoothing function form, X represents a climatic factor and d represents the degree of freedom of the smoothing function for the independent variable. Climatic factors indirectly impact dengue transmission by influencing the biological functions of the mosquito vector, resulting in a lag effect (Ramachandran et al., 2016).

Melinda K. Butterworth et al. utilized predicted climate conditions derived from a global climate model (GCM), and constructed a dynamic mosquito simulation model (DyMSiM) based on climate data from at 23 sites in the southeastern United States. The findings revealed the potential spread of dengue in various locations within the region. The highest risk for dengue transmission occurred during summer months (July-September), while no cases were reported in winter. However in Florida and Texas, dengue transmission was also possible in spring and autumn. It was hypothesized that the prolongation of the extrinsic incubation period (EIP) at lower temperature inhibited the spread of dengue (Butterworth et al., 2017). It should be noted that the imported cases were not taken into consideration in the above researches. Nonetheless, another research conducted in Florida demonstrated that temperature also influenced the number of imported dengue cases (Stephenson et al., 2022).

Dengue cases over a 19-year period (January 1997 to December 2015) were collected monthly in East Delhi, India. Correlation analyses were conducted to examine the relationship between dengue cases and climatic conditions including rainfall, temperature and humidity. Four prediction models were developed using a negative binomial generalized linear model. Among them, rainfall, temperature and humidity served as independent variables, while dengue cases serve as the dependent variable. The prediction models were constructed for the same month as well as lags of one, two and three months as climatic factors. The result showed that the model with a two-month lag provided the best prediction of dengue epidemics. Additionally, the EIP of dengue virus was shortened at 30°C, which may facilitate the spread of dengue (Ramachandran et al., 2016). Analysis of seasonal patterns of average maximum and minimum temperatures for dengue cases revealed that reported cases peaked between August and October during 2015 to 2018, which corresponded to the months following the highest and lowest temperatures recorded. The temperature at peak of dengue was between 25°C and 27°C (Singh and Chaturvedi, 2022).

Data on cases of dengue hemorrhagic fever (DHF) were collected alongside the temperature and humidity measurements in Menado, Indonesia. The correlation between temperature or humidity and DHF incidence was analyzed using the Spearman’s rank correlation test. The results showed that the highest temperature occurred in August at 28.7°C, while the lowest cases of DHF were observed in September. Conversely, the lowest temperature occurred in February at 25.9°C, while the highest cases of DHF were reported in January. These findings indicate a significant correlation between dengue prevalence and temperature (Monintja et al., 2021). Another study conducted in Makassar, Indonesia employed the generalized estimating equations method (GEE) to analyze the correlation between dengue cases and climate, which presented a significant negative correlation (Susilawaty et al., 2021). Given that Indonesia is located in a tropical region with high average annual temperatures, the reduced survival rate and daily activity of mosquitoes could potentially decrease the dengue transmission.

In China, daily dengue cases were collected in Guangdong Province from 2005 to 2015. Temperature and precipitation data were obtained from the China Meteorological Data Sharing Service. A zero-inflated generalized additive model (ZIGAM) was constructed on the basis of the GAM to analyze the trend of dengue incidence in relation to mosquito densities. Results indicated a positive effect of temperature on the incidence of dengue (Xu et al., 2017). Another study revealed that the effect of mean(28°C), minimum(23°C) and maximum(32°C) temperatures on dengue was non-linear (Wu et al., 2018).

## Temperature changes the distribution of *Aedes* mosquitoes

### Effects of temperature on the biological characteristics of *Aedes* mosquitoes

Temperature plays a vital role in the development and survival of *Aedes* mosquitoes. The optimal temperature range for their development is 25°C-30°C. When the temperature exceeds 40°C, adult mosquitoes die, and eggs and larvae fail to develop (Reiskind and Zarrabi, 2012). Additionally, both *Ae.albopictus* and *Ae.aegypti* larvae are unable to development at 10°C (Reiskind and Zarrabi, 2012; Marinho et al., 2016). *Ae.albopictus* adults do not feed on blood, while the eggs and larvae need a minimum temperature of 15°C for development (Reiskind and Zarrabi, 2012).

At moderate temperature (20°C-30°C), temperature does no significantly affect the survival of *Aedes* mosquitoes (Alto and Bettinardi, 2013).Regardless of the rearing temperature of the larvae, the survival rate of adult mosquitoes is significantly higher at 20°C compared to other temperatures (Alto and Bettinardi, 2013). Generally, an increasing temperature is accompanied by an increased activities in *Aedes* mosquitoes, but excessively high temperatures can shorten their lifespan and reduce their population size (Myer et al., 2020). Females *Ae.albopictus* tend to survive longer than males at different temperatures. As the temperature rises, the life cycle of *Ae. albopictus* shortens, and the population growth rate increases (Reiskind and Zarrabi, 2012). *Ae.aegypti* exhibits a greater tolerate to temperature variations than *Ae. albopictus*, which provides a competitive advantage (Myer et al., 2020). *Ae.aegypti* struggles to survive at extremely high or low temperatures, such as below 11°C or above 36°C (Iwamura et al., 2020). *Ae.aegypti* is less mobile and unable to feed on blood below 14°C-15°C, leading to its mortality. It also cannot survive for more than 2-3 days without a blood meal at tropical temperature (Alto and Bettinardi, 2013).

Temperature affects characteristics of adult *Aedes* mosquitoes. During the developmental stage, changes in rearing temperature affect the external traits, such as the wing length and the adult size. Higher temperatures(24°C-29°C) and sufficient food are beneficial to mosquito’s ingestion, resulting in shorter wings and heavier weight in *Aedes* mosquitoes. In contrast, lower temperatures(14°C-19°C) and insufficient food lead to mosquitoes with longer wings and lower weight (Reiskind and Zarrabi, 2012; Alto and Bettinardi, 2013).

Temperature also affects the reproduction activity of *Aedes* mosquitoes. For example, in areas with an average annual temperature of 22°C, the weekly production of *Aedes* mosquito eggs is high when the Daily Mean Temperature Range(DTR)ranges from 12°C to 18°C, but it decreases when the temperature exceeds 18°C (Betanzos-Reyes et al., 2018). Extreme temperature like more than 36°C could greatly reduce the number of eggs production (Marinho et al., 2016).

### Effects of temperature on *Ae.albopictus* distribution

*Ae.albopictus* is native to Southeast Asia and has recently expanded its range to Africa, where it was first reported in 1990 in South Africa (Cornel and Hunt, 1991). Over the past decade, *Ae.albopictus* had spread to several Central African countries (Paupy et al., 2009). It rapidly pullulated in Nigeria in 1991 (Savage et al., 1992) and appeared in Central Africa in 2000 (Kamgang et al., 2010).

Temperature exerts an influence on the distribution of *Ae.albopictus*. It became active when the temperature rises above 13°C, and its population gradually increases following the risen temperature. When the temperature exceeds 36°C, the population began to decline. *Ae. albopictus* is present in most Asian cities and large parts of the America (Kraemer et al., 2015). For example, in Brownsville, Texas, *Ae. albopictus* populations generally increases before March and after August, reaching its peak in winter and decreasing in spring and summer (Brady et al., 2014). *Ae. albopictus* is well adapted to northern South America, where diurnal temperature fluctuates significantly (Brady et al., 2014). In Portugal, *Ae.albopictus* becomes active in May, with an average minimum temperature of over 13°C and an average maximum temperature of 26.2°C. The peak abundance of *Ae. albopictus* populations occurs between September and November (average temperatures around 23°C) (Osorio et al., 2020). In China, *Ae.albopictus* can be found in the southern, eastern, and central regions, and even in some parts of northeastern China. Compared to *Ae.aegypti, Ae.albopictus* is more adaptable to diurnal and seasonal temperature differences. As temperature increases in the future, *Ae.albopictus* populations will increase in the central and northern regions, where diurnal temperature differences are more remarkable. Additionally, the warmer south will continue to provide a suitable habitat for *Ae.albopictus* (Liu B et al., 2019).

### Effects of temperature on *Ae.aegypti* distribution

*Ae.aegypti* was originally believed to have originate from Africa (Brown et al., 2014). In 2000, indigenous mosquito species *Ae.aegypti* were found in Central Africa (Kamgang et al., 2010). Moreover, a study noted that *Ae. aegypti* can survive in cold winter and will probably spread to colder areas driven by climate change(Kramer et al., 2021).

The distribution of *Ae.aegypti* is strongly influenced by temperature (Dickens et al., 2018).There is a positive correlation between *Ae.aegypti* populations and minimum temperatures (Li et al., 2019). Areas with higher minimum temperature (>8°C) are more favorable for the survival of *Ae.aegypti* (Dickens et al., 2018). When the minimum temperature ranges between 16°C and 20°C, mosquito populations are larger than the average. However, when the minimum temperature exceeds 20°C, *Ae.aegypti* populations are not affected by further temperature changes. Adults *Ae.aegypti* populations display a seasonal pattern, with low densities in winter and high densities in summer (Li et al., 2019). Due to their temperature dependence tropical and subtropical areas are their main distribution areas. The temperature differences between day and night are low in Portugal, Spain, southern France, and coastal Italy, which are favorable for the survival of *Ae. Aegypti* (Dickens et al., 2018). In China, the habitat of *Ae. aegypti* is confined to specific regions with an annual mean temperature above 20°C, such as Hainan Province, southern Guangdong Province, southern Yunnan Province, et al. When temperatures rise above 35°C, the habitat of *Ae. aegypti* is adversely affected (Liu B et al., 2019). The abundance of *Ae. aegypti* gradually increased in all regions from July to October, with a peak in August (Liu B et al., 2019).

### Temperature alters the structure of dengue virus

DENV belongs to Flaviviridae family of flavivirus. Based on antigenicity difference DENV can be divided into four serotypes(DENV-1, DENV-2, DENV-3, DENV-4) (Sharma et al., 2019). Cross-antigenicity exists among different types of DENV. The structure of DENV-2 is similar to DENV-1 and DENV-3, but exhibits lower similarity with DENV-4 (Lok et al., 2008). DENV is single positive-stranded RNA virus, which encodes three structural proteins and seven nonstructural proteins. The structural proteins include the capsid (C), membrane (M) and envelope (E) protein. The E protein which forms ninety dimers on the surface of mature DENV, plays a significant role in the process of pathogenicity and immunity (Lim et al., 2017a; Boigard et al., 2018; Sharma et al., 2019). The E protein consists of three domains, E-DI [residues 1-52; 132-193; 280-296], E-DII [residues 53-131; 194-279] and E-DIII [residues 297-394]. D-III demonstrates variability of different serotypes as the site of initial interaction with cellular receptors (Slon et al., 2017; Sharma et al., 2019). The seven nonstructural proteins involve NS1\NS2a\NS2b\NS3\NS4a\NS4b\NS5, are involved in viral replication, protein processing, and the assembly and release of viral particles (Boigard et al., 2018).

Temperature mainly affects the structure of the E protein, thereby impacting the overall structure of DENV (Lim et al., 2017b). DENV-2 displays a smooth surface at 28°C, and 96% of them became bumpy at 37°C. The temperature-induced structural alterations usually occurred between 31°C and 35°C and are irreversible, as no structural change are observed when the temperature is lowered from 37°C to 4°C (Zhang et al., 2013). The soluble recombinant E (sRecE) protein is similar to the conformation of E dimers displacing on the virion surface. The sRecE protein of DENV-2, DENV-3 and DENV-4 was in equilibrium between dimer and monomer. At 23°C, sRecE of DENV-2 exists as a dimer, while that of DENV-3 and DENV-4 exists as monomers. At 37°C, sRecE of DENV-2, DENV-3, and DENV-4 mainly exists as monomer (Kudlacek et al., 2018). Another study also demonstrated that the structure of E protein of DENV-2 is irreversible from 40°C to 25°C., with a 50-fold decrease in the ability to form E-dimers at 40°C comparing to 25°C (Sharma et al., 2019). Therefore, the weakened ability of E protein to form dimers may be responsible for the irreversible structural alteration of DENV-2. On the contrary, the structural changes of E protein in DENV-1 are reversible (Sharma et al., 2019).

Virulence of DENV is independent of structural transition but is correlated with intrinsic dynamics. When BHK21 cells were infected with DENV-2, the number of plaque declined by 3-fold at 40°C compared to 25°C and 37°C. Such decrease was caused by the flexibility loss in E-DIII of DENV-2 rather than the structural changes. The flexibility loss in E-DIII of DENV-2 may inhibit the interaction between E-DIII and host cells, thereby further reducing viral infectivity (Sharma et al., 2019). In C6/36 cells infected with DENV-2, the titer of viral particles at 37°C was higher than that at 28°C (Pandey et al., 1998). In AG129 mice infected with DENV-2 incubated at different temperatures has many differences. The mice infected DENV-2 incubated at 39°C died more rapidly than incubated at 28°C.The former have more serious organ injures (Modak et al., 2023).

## Temperature affects the vector competence of *Aedes* mosquitoes

### Vector competence of mosquitoes

Vector competence is the ability of mosquitoes to become infected with pathogens and then transmit them to new hosts (Souza-Neto et al., 2019). Various biological and environmental factors could affect vector competence in mosquitoes. Biological factors include mosquito species and strains of virus, while environmental factors include climate, water sources and insecticides (Lounibos and Kramer, 2016; Michael, 2018; Ruckert and Ebel, 2018; Ingham et al., 2021).

The interaction between viruses and mosquitoes is a multi-factorial phenomena, which is determined by both virus strains and mosquito genotypes. MOYO-S and MOYO-R were two groups of *Ae.aegypti.* MOYO-R was difficult to treat dengue infection, while MOYO-S was susceptible. A large number of genes were differentially expressed between MOYO-S (susceptible) and MOYO-R (refractory) strains of *Ae.aegypti* infected with DENV-2. The results suggested that susceptibility to DENV-2 is associated with structural/evolutionary features of the responsive genes in MOYO-S/MOYO-R strains (Behura and Severson, 2012). The vector competence of *Aedes* mosquitoes may vary depending on the virus strain. For instance, the Southeast Asian genotype (SEA strain) of DENV-2 could spread faster than the American genotype (AM strain) (Anderson and Rico-Hesse, 2006). Additionally, the vector competence of *Aedes* mosquitoes is related to the viral titer in blood meal, with a higher titer facilitating virus transmission (Van den Eynde et al., 2022).

Temperature plays a significant impact on the vector competence of *Aedes* mosquitoes. Optimal temperatures for DENV transmission are typically between 20°C and 26°C (Ciota et al., 2018). Temperature affects various aspects of mosquito biology, including egg hatching rates, larval developmental time, and adult survival rates, which, in turn, influence vector competence. When survival rate increased from 0.80 to 0.95, the number of potential transmissions increased fivefold. An increase in temperature of 10°C led to a halving of the bite interval and increased transmission by at least 2.4-fold (Barbazan et al., 2010). The rearing environment of mosquito larvae also affects vector competence, as the presence of diverse microorganisms in the breeding water could affect the ability of *Ae.aegypti* to transmit virus (Louie and Coffey, 2021). Adult female *Aedes* mosquitoes acquire nutrients from nectar and carbohydrates in the blood, which mainly derived from sugars, including sucrose, fructose and glucose (Elina and Matthew, 2020). The sugar diet of *Aedes* mosquitoes may reduce their vector competence. Sugar intake could increase the expression of antiviral genes in the digestive tract of female mosquitoes, thereby blocking the initial infection and dissemination of viruses (Almire et al., 2021). In addition, vector control is the most effective and economical method to prevent and control mosquito-borne diseases by reducing the vector competence of mosquitoes through direct killing (Ingham et al., 2021).

Vector competence of mosquitoes is closely associated with immune pathways and tissue barriers (Gloria-Soria et al., 2017). The immune pathways in mosquitoes primarily include RNA interference (RNAi), Toll, immune deficiency(IMD), and Janus kinase/signal transducer and activator of transcription (JAK/STAT) (Liu et al., 2018; Lan et al., 2022). Additionally, Phenoloxidase (PO) plays a crucial role in insect immunity as a key enzyme for melaninization, which is responsible for mosquitoes’ defense against pathogens (Liu et al., 2018; Ji et al., 2022). Arboviruses must overcome several barriers in mosquitoes, including the midgut infection barrier (MIB), midgut escape barrier (MEB), salivary gland infection barrier (SGIB), and salivary gland escape barrier (SGEB) (Franz et al., 2015). The midgut acts as the initial barrier to prevent virus transmission, and the mosquito’s immune system begins to suppress the virus in this region (Liu et al., 2018).

### Effects of temperature on the vector competence of *Ae. albopictus*

The vector competence of *Ae. Albopictus* is generally lower than that of *Ae. aegypti.* However, *Ae.albopictus* become a main vector for the transmission of DENV in certain regions due to its widespread distribution. When the temperature falls below 18°C, *Ae.albopictus* does not transmit DENV but can transmit chikungunya virus (Wimalasiri-Yapa et al., 2019). The ability of *Ae.albopictus* to transmit DENV increased as the temperature risen between 18°C and 32°C. However, when the temperature exceeded 32°C, the mortality rate of *Ae.albopictus* increased, potentially reducing its vector competence (Liu et al., 2017).

**Figure 1:**
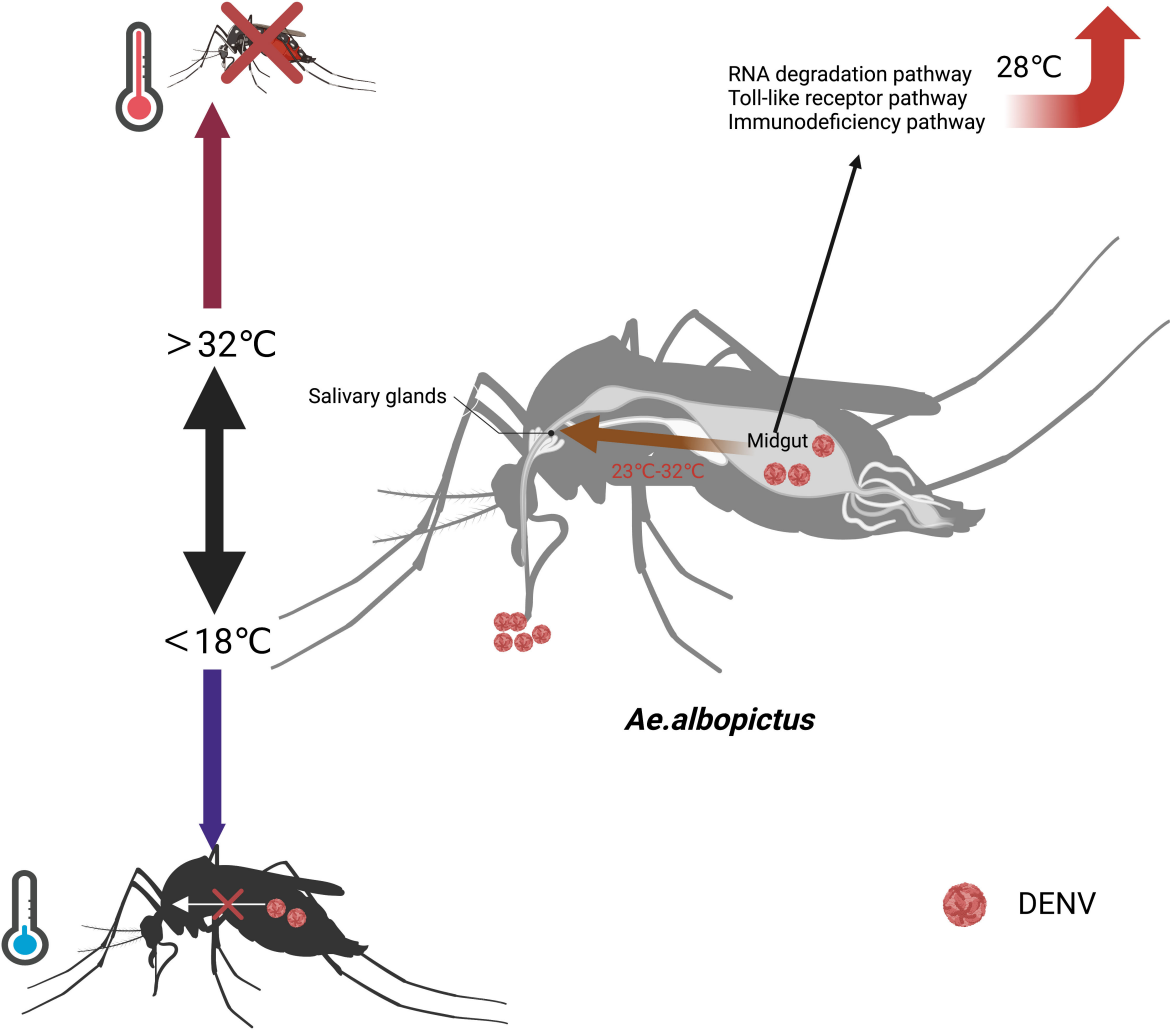
Effects of temperature on the vector competence of *Ae. albopictus*. When the temperature exceeds 32°C, the mortality rate of *Ae.albopictus* increases. When the temperature falls below 18°C, *Ae.albopictus* does not transmit DENV. At 18°C, DENV is localized to the midgut of *Ae.albopictus* and proliferate.DENV breaks through the midgut barrier and invades the salivary glands of *Ae.albopictus* between 23°C and 32°C. At 28°C, the RNAi pathway, Toll pathway, and IMD pathway of the midgut in *Ae. albopictus* are enhanced.

The mechanism by which temperature affects the vector competence of *Ae. albopictus* had not been fully clarified. Studies suggest that higher temperatures shorten the gonotrophic cycle and led to frequent blood feedings, thereby increasing mosquito’s vector competence (Martens et al., 1995). Temperature also affects the virus to across the midgut barrier. DENV-2 was localized to the midgut of *Ae.albopictus* and slowly proliferated at 18°C. However, DENV-2 broke through the midgut barrier and invaded the salivary glands of *Ae.albopictus* between 23°C and 32°C (Liu et al., 2017). The RNAi pathway, Toll pathway, and IMD pathway of the midgut in *Ae. albopictus* were enhanced at 28°C. The key genes regulated by temperature included heat shock protein 70(HSP70), CCR4-NOT complex, and Myeloid differentiation primary response protein 88 (MyD88) (Liu et al., 2022). HSP70 was the most critical component for DENV-4 entering C6/36 cells (Vega-Almeida et al., 2013). The mRNA expression in the HSP70 was regulated upwards at 37°C and downwards at 39°C (Sivan et al., 2017). The expression level of CCR4-NOT complex gene was upregulated in DENV-2 infected cells, which was conducive to the proliferation of DENV. At 32°C, the CCR4-NOT complex gene is highly expressed in DENV-2 infected cells, facilitating the proliferation of DENV-2 and its ability to break through the midgut barrier (Liu J et al., 2019). However, further functional validation of these key factors is needed in the future (**Figure 1**).

### Effect of temperature on the vector competence of *Ae. aegypti*

*Ae.aegypti* could transmit DENV between temperatures of 22°C and 32°C, but it couldn’t survive when the temperatures rose to about 40°C (Marinho et al., 2016). High mortality rates of mosquitoes inhibited the spread of DENV, thereby reducing their vector competence (Chepkorir et al., 2014). Significant temperature differences between day and night could influence the vector competence of *Ae.aegypti* by changing vector vital signs and shortening lifespan, which in turn reduced the midgut infection rate and transmission rate for DENV-1 and DENV-2 (Lambrechts et al., 2011). It is worth noting that the impact of temperature fluctuations on vector competence differs based on average temperature levels. At lower average temperatures (<18°C), a temperature fluctuation of 6.26°C between day and night increased the ability of *Ae.aegypti* to infect and transmit DENV-1. However, at higher average temperatures (≥18°C), the same temperature fluctuation between day and night reduced the vector competence of *Ae. Aegypti* (Carrington et al., 2013).

**Figure 2:**
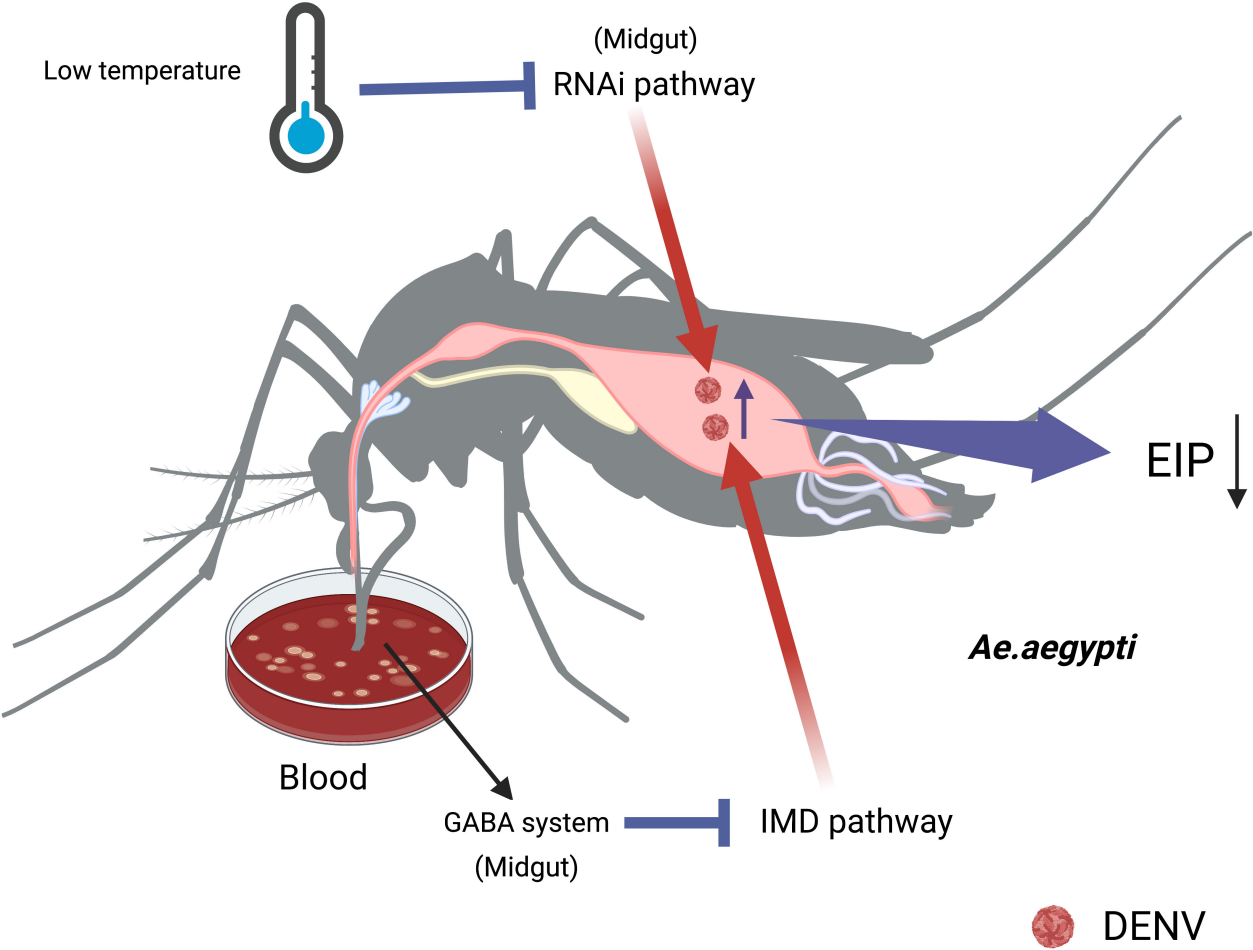
The temperature affects the midgut’s immunity to change the vector competence of *Ae.aegypti*. The impairment of RNAi pathways increases DENV titers in midgut at low temperature, making the viruses easily disseminate to other tissues and shorten the EIP. Sucking blood activates GABA associated system, enhancing DENV replication in the midgut of *Ae.aegypti* by inhibiting the IMD pathway.

The mechanism that how temperature affects the transmission of DENV by *Ae. aegypti* has not been fully elucidated. The RNAi pathway of *Ae.aegypti* was compromised at low temperatures, making mosquitoes from warmer regions more susceptible to virus at cold temperatures compared to those from colder regions (Gloria-Soria et al., 2017). Moreover, the impairment of the RNAi pathway in *Ae. aegypti* increased DENV-2 titers in the midgut, facilitating the dissemination of viruses to other tissues and shortening the EIP (Franz et al., 2015). *Ae. aegypti* infected with DENV-2 is regulated by the siRNA pathway. It can accelerate DENV replication and shorten EIP by silencing of Dcr 2 or Ago 2 (Sanchez-Vargas et al., 2009). Additionally, *Ae.aegypti* activated γ-aminobutyric acid (GABA) associated system through blood feeding, enhancing the DENV-2 replication by inhibiting the IMD pathway (Luplertlop et al., 2011). (**Figure 2**)

We refer to other flaviviruses such as Zika virus (ZIKV) and chikungunya virus to indirectly reflect the possible mechanism that how temperature affects the transmission of DENV by *Ae.aegypti*. The immune reaction of *Ae. aegypti* infection with the virus could be divided into four components: recognition of pathogen, activation of signal pathway, immune response and immune regulation (Etebari et al., 2017). The recognition of the pathogen was depended on pattern recognition receptors (PRRs) (Julián, 2016), including the clip-domain serine proteases (CLIPs) family B(Wang and Wang et al., 2021), the leucine-rich repeats protein(LRR) (Zhao et al., 2019), thioester-containing protein (TEP) (Weng et al., 2021), and galectins (Xiaohua et al., 2020). The immune response of *Ae. aegypti* infected with ZIKV was strongest at 28°C, as evidenced by upregulated Dicer-2 activity and the strengthened immune pathways including the Toll pathway, IMD pathway, and JAK/STAT pathway. However, these immune response weakened at 32°C (WimalasiriYapa et al., 2021). Melaninization, which plays a role in the *Aedes* mosquitoes’ defense against viral infections, is affected by temperature. At 20°C, phenoloxidase and C-type lectin were upregulated in the midgut of *Ae. aegypti*, reducing its vector competence to transmit ZIKV (Murdock et al., 2012).

**Figure 3:**
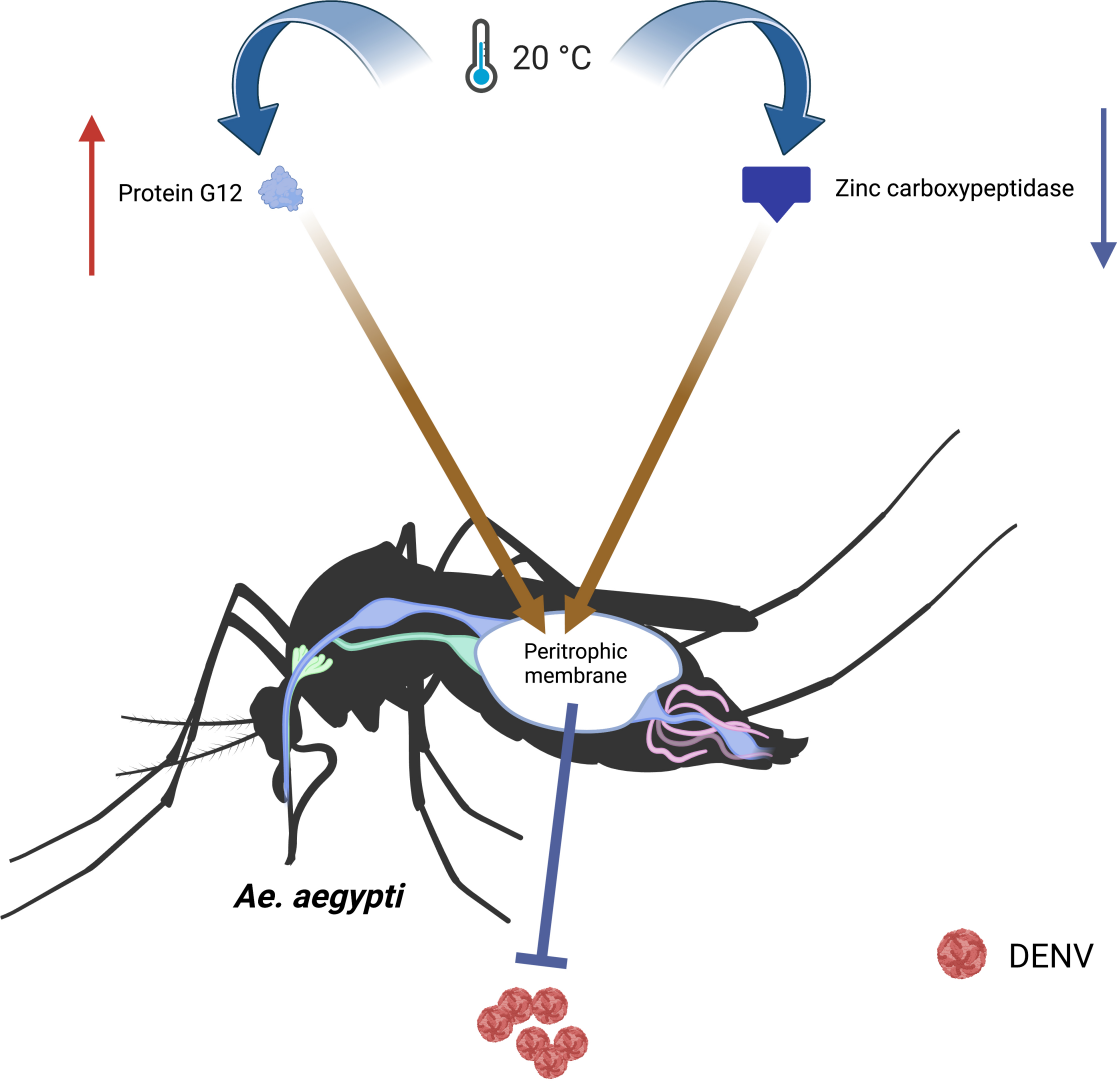
The temperature affects the metabolism to change the vector competence of *Ae.aegypti*. At 20°C, the protein G12 involved in blood meal digestion and nitrile-specific detoxification increases, and the zinc carboxypeptidase involved in blood meal digestion significantly downregulates, which lead to form a PM. The PM slows down the spread of pathogens and reduces vector competence.

The temperature could affect the vector competence of mosquitoes through altering their metabolism. The biochemical activity of mosquitoes was impaired at low temperatures, resulting in the accumulation of fat and reduced energy reserves (Angilletta et al., 2010). The digestion of blood meal in the *Ae.aegypti* is slow under low temperature. Zinc carboxypeptidase involved in blood meal digestion was significantly downregulated at 20°C to form a peritrophic membrane (PM), which could protect the midgut against pathogens (Ferreira et al., 2020). Additionally, the protein G12 involved in blood meal digestion and nitrile-specific detoxification was increased at 20°C. β-galactosidase and α-N-acetylgalactosaminidase are two digestive proteases involved in glycoside hydrolysis. These highly induced enzymes and proteins contribute to the formation of the PM, slowing down the spread of pathogens and reducing the vector competence of mosquitoes(Santamaria et al., 2015; Ferreira et al., 2020). (**Figure 3**)

## Future directions

Previous researches have demonstrated that temperature is closely related to the transmission and epidemic of dengue. However, the detailed mechanism and specific targets are still unclear. Although *Ae. albopictus* and *Ae. aegypti* share similar ecological habitats the distribution is different. The growth, development and survival of *Aedes* mosquitoes are influenced by fluctuant temperatures. Whole-genome sequencing results can provide valuable insights for further research. The genome of *Ae.albopictus* comprising 1,967 Mb, is the largest mosquito genome sequenced to date, and its size results principally from an abundance of repetitive DNA classes (Chen et al., 2015). *Ae. aegypti*, on the other hand, lacks heteromorphic sex chromosomes and its genome size was estimated to be 813 Mb (Nene et al., 2007). The difference in genome sequence may contributes to the difference of traits between these two *Aedes* species. The structure of DENV could affected its virulence and targeted in antiviral strategies (Sharma et al., 2019). It resulted from the large-scale conformational changes and intrinsic dynamics of DENV E proteins influenced by the temperature. The dynamic conformations of the same virus at different temperatures and crystal structures of different virus types need to be further improved. In addition, the mechanisms by which temperature affects the vector competence of *Aedes* mosquitoes to transmit DENV need to explore. The immune pathways and factors of *Aedes* mosquitoes have been changed after infection with DENV under different temperatures, the function of key immune molecule should be further clarified. The CRISPR-Cas9 system served as a genome-engineering tool offers a new perspective on the antiviral mechanisms of *Aedes* mosquitoes (Kistler et al., 2015; Adelman and Tu, 2016). The changes of vector competence of *Aedes* mosquitoes might be analyzed by constructing over-expressed plasmids or using CRISPR-Cas9 system in combination with microinjection technique. These approaches provide guidance for the prevention and control of dengue.

## Author contributions

ZL designed the framework of this manuscript. QZ, LL, JH, JG, ZW, YH, ZX contributed to writing and editing of this manuscript. ZL, YL, TL, QZ, LL contributed to the literature review and editing of this manuscript. All authors contributed to the article and approved the submitted version.
