# Corrigendum: The effect of temperature on dengue virus transmission by *Aedes* mosquitoes

**DOI:** 10.3389/fcimb.2023.1320461

**Published:** 2023-10-24

**Authors:** Zhuanzhuan Liu, Qingxin Zhang, Liya Li, Junjie He, Jinyang Guo, Zichen Wang, Yige Huang, Zimeng Xi, Fei Yuan, Yiji Li, Tingting Li

**Affiliations:** ^1^ Department of Pathogen Biology, Center for Tropical Disease Control and Research, School of Basic Medical Sciences and Life Sciences, Key Laboratory of Tropical Translational Medicine of Ministry of Education, Hainan Medical University, Haikou, China; ^2^ Department of Pathogen Biology and Immunology, Jiangsu International Laboratory of Immunity and Metabolism, Jiangsu Key Laboratory of Immunity and Metabolism, Xuzhou Medical University, Xuzhou, China; ^3^ School of Imaging Medical Sciences, Xuzhou Medical University, Xuzhou, China

**Keywords:** temperature, dengue virus, *Aedes albopictus*, *Aedes aegypti*, vector competence

In the published article, there was an error regarding the affiliation for author Zhuanzhuan Liu. As well as having affiliation 1, they should also have “*Department of Pathogen Biology, Center for Tropical Disease Control and Research, School of Basic Medical Sciences and Life Sciences, Key Laboratory of Tropical Translational Medicine of Ministry of Education, Hainan Medical University, Haikou, China”*. The new affiliation should be the first of all affiliations, and the affiliation numbers for authors Yiji Li and Tingting Li should be changed from 3 to 1. The final version of authors and affiliations are as shown above.

The authors apologize for this error and state that this does not change the scientific conclusions of the article in any way. The original article has been updated.

